# Is Connected Health Contributing to a Healthier Population?

**DOI:** 10.2196/jmir.8309

**Published:** 2017-11-10

**Authors:** Carmen G Loiselle, Saima Ahmed

**Affiliations:** ^1^ Department of Oncology Faculty of Medicine McGill University Montreal, QC Canada; ^2^ Ingram School of Nursing Faculty of Medicine McGill University Montreal, QC Canada; ^3^ Segal Cancer Centre Jewish General Hospital Montreal, QC Canada; ^4^ Experimental Medicine McGill University Montreal, QC Canada

**Keywords:** connected health, mHealth, eHealth, uHealth, ubiquitous health

## Abstract

Connected health tools, including mobile phones, incorporate various functions that capture events, direct actions, and make informed decisions based on complex sources of data. Connected health, a term recently proposed by some academics and industry, refers to the development, testing, and integration of smart technology tools into health care. Through these means, connected health creates interconnectivity across various environments, profoundly changing the way we learn, self-regulate, and communicate with one another. In health care, mobile phones enable more precise diagnostics, personalized health recommendations that enhance patient experiences and outcomes while containing health care costs. However, for connected health to achieve its full potential, issues must be addressed pertaining to active engagement in use, privacy, security, and quality, as well as the development of evidence-based guidelines. This commentary discusses these key challenges and explores the promise of connected health, specifically eHealth and mHealth. Anchored within the context of cancer, the authors’ area of expertise, the ideas put forward can readily be applied to other health-related disciplines.

Our world is increasingly interconnected thanks largely to the technology behind tools that link us, gather information, and display data. This profoundly changes the way we learn, connect, and regulate various aspects of the self (eg, physical, psychological, social, and occupational domains). Globally, more than 50% of the world's population owns a mobile device [1]. In the developed world, this number rises to almost 90%, a clear indicator of the significant impact that science and technology have had on our daily lives [1].

Connected health, a newish term recently proposed by some academics and industry, refers to the development, testing, and integration of smart technology tools into health care [2]. When it comes to physical, psychological, or other health-related issues, connected health can drastically change how health information and support are accessed, communicated, monitored, and acted upon. Mobile health (mHealth) apps are being positioned by developers at the very core of convenience and capabilities, allowing with 1-click easy access to our dearest curiosities, needs, worries, and wants. In 2015, there were more than 165,000 mHealth apps available for users to download [3]. Recent reports suggest that these can significantly support patient engagement, self-management, and empowerment [2]. Interestingly enough, however, recent statistics in the United States indicate that merely 12% of health-related apps are responsible for 90% of downloads [3]. One may ask then—why is the wide range of health apps available so underused given significant trends toward fitness and health? What may be important barriers that potential users face? A recent study examining user perceptions found that lack of health app literacy and not knowing about the various options available were the two most important barriers to adoption [4]—this is even more so for seniors, lower socioeconomic groups, those with less than a high school education, and non-English speakers [2,3]. From a technological point of view, the current myriad of connected health options (eg, health apps) makes it difficult for consumers to choose the most personally relevant and high-quality tools. In the oncology field, the options can be overwhelming to the millions of individuals affected—Belong–Beating Cancer Together, Best Cancer Fighting Foods, Breast Cancer Reference, Cancer Quotes, Cancer Therapy Adviser, ePrognosis, iCancerHealth, Kill The Cancer, MyCancerCoach, My Cancer Manager, and more.

While others point to the paucity of decision aids to inform optimal choice and lack of evidence for significant effects [3], we contend there are more proximal reasons for connected health underuse. The current focus may be overly placed on technology while ignoring the need for a true paradigm shift in health care—one that welcomes, encourages, and requires patients to be activated and engaged in their own wellness and care. In addition, uptake strategies must be in place to ensure that individual engagement in connected health is highest prior to facing health and illness issues. Perhaps a better understanding of the root causes, historically, of the health literacy gap would shed further insight into this important factor. In addition, the prescreening of high-quality apps with hands-on guidance, especially for those less likely to use connected health, is known to increase adoption and maintenance [5]. More engaging features include perceived usefulness, simplicity, and credibility of the sources [4,5]. Therefore, knowledgeable and credible health care providers are in an excellent position as promoters and facilitators of connected health use [6,7,8]. In line with a change in paradigm, there must be a shift in the role of health care providers from fixer to coach, enabling empowerment as much as possible in people facing health challenges, whether acute or chronic.

In terms of quality control, consumers (and many manufacturers) remain unaware of technological standards and regulations to which connected health products must be held accountable [9]. The manner in which app stores are set up, for instance, means that popular apps (but not necessarily the best ones) are more visibly displayed and more readily downloadable. However, lack of supporting evidence means that users often rely solely on subjective 5-star ratings to gauge the quality of apps. Whereas recent frameworks have been put forward to assess connected health quality, such as the Reach, Effectiveness, Adoption, Implementation, and Maintenance (RE-AIM) framework to evaluate health behavior interventions, the Mobile App Rating Scale (MARS) to access quality, Consolidated Standards of Reporting Trials (CONSORT-EHEALTH) for the standardized reporting of mHealth and electronic health (eHealth) research, and the upcoming Canadian Institutes of Health and Research guidelines [9-15], one cannot underestimate the importance of bringing people to become more acquainted with connected health (ie, increasing the number of users) before devoting too many resources to developing and ranking tool quality—tools that individuals may not even use. With increased adoption over time, consumers will also become savvier in discriminating among the various options available to them.

With regard to eHealth dissemination, the transmission of health-related knowledge over the Internet, misinformation can also be an important issue, with little attention paid to verifying the trustworthiness and timeliness of the information [16,17]. Apple’s App Store warns that “medical apps that could provide inaccurate data or information, or that could be used for diagnosing or treating patients may be reviewed with greater scrutiny” [18]. Apps that offer peer-to-peer support with content that is user-generated may be particularly at risk for errors and misinformation. While community and support needs may be readily met, anecdotal accounts are not reliable sources and have been associated with negative effects on health outcomes, health care use, and health care provider-patient relationships [16,19]. Government organizations, associations, and the private sector have begun countering this by creating content on their own that provides relevant, trustworthy, and high-quality information [16].

As we are well aware, health care information is particularly sensitive to privacy and security issues, and health care providers are particularly worried about this [3,20,21]. Whereas most oncologists, for instance, know that patients rely on connected health for cancer information and support, they are uncomfortable recommending particular apps to patients [22]. To this end, the National Institutes of Health Informatics, serving as a link among academia, the health informatics industry, and users, offers a variety of resources as a means of guiding innovation uptake and dissemination.

For specific privacy and security issues, concerned consumers are urged to read privacy statements before using connected health to have more control of their privacy [23]. However, regulations vary widely from country to country and, as of yet, no international standards exist [24]. The US Food and Drug Administration, for example, only regulates “a small subset of mobile medical apps that may impact on the performance or functionality of currently regulated medical devices” [25]. In contrast, the European Commission put forward new legislation as part of their ePrivacy Directive and General Protective Regulation of Personal Data [26]. The Department of Justice will officially enact the regulation on May 25, 2018. It will apply to all computers, mobile phones, and tablets and will require informed consent by users for cookies, a form of Web-based data storage, as well as reporting from Internet and app providers of any personal data breaches [27]. Recently, the addition of blockchain technologies that remove the characteristic of infinite reproducibility of data has drastically reduced worries about data security. Benefiting both institutions and patients alike, they remove the onus to safeguard sensitive health data while facilitating the sharing of data. Developers of patient portals and patient navigation platforms are now relying on such security technology [28].

Ultimately, the large number and varying quality of apps available today—combined with few evidence-based guidelines—undermine connected health uptake. However, universally activating people to own a role in their health, wellness, and care as well as empowering them through ready access to relevant connected technologies will translate into unleashing connected health potential like never before. Through the creation of a relevant repository of personal data, individuals will be empowered to make more informed health and lifestyle decisions and changes [29].

*Editorial Note:* JMIR Publications discourages the use of "cHealth" (connected health) as it is not clear whether and how the term differs from eHealth, mHealth or uHealth (ubiquitous health).

## Abbreviations

RE-AIM: Reach, Effectiveness, Adoption, Implementation, and Maintenance
MARS: Mobile App Rating Scale
CONSORT-eHEALTH: Consolidated Standards of Reporting Trials - eHealth extension
